# Congenital Temporomandibular Joint Ankylosis: Case Report and Literature Review

**DOI:** 10.1155/2016/5802359

**Published:** 2016-04-13

**Authors:** Ryan Chin Taw Cheong, Karim Kassam, Simon Eccles, Robert Hensher

**Affiliations:** ^1^Colchester Hospital University NHS Foundation Trust, Turner Road, Colchester, Essex CO4 5JL, UK; ^2^Northwick Park Hospital, Watford Road, Harrow, Middlesex HA1 3UJ, UK; ^3^Chelsea and Westminster Hospital, 369 Fulham Road, London SW10 9NH, UK; ^4^Lister Hospital, Chelsea Bridge Road, London SW1W 8RH, UK

## Abstract

Congenital temporomandibular joint (TMJ) ankylosis is an uncommon condition that presents itself at or soon after birth in the absence of acquired factors that could have contributed to the ankylosis such as infection and trauma. The experience of managing one such case is reported in light of a review of the literature on this condition. Key management principles include adequate removal of the ankylotic mass, costochondral grafting, and post-op physiotherapy. Most patients reported in the literature with the condition experienced relapse. This echoes our own experience where there was recurrence of the ankylosis. However, after removal of the ankylotic mass, the patient maintains a satisfactory maximal incisal opening (MIO) till the present day. The additional challenges faced in the congenital form in addition to the already complex management of acquired paediatric temporomandibular joint ankylosis are (1) much earlier insult to the TMJ, (2) reduced opportunity for neuromuscular development of the muscles of mastication, and (3) reduced compliance with postoperative physiotherapy programmes due to the younger age of these patients.

## 1. Introduction

Congenital temporomandibular joint (CTMJ) ankylosis was first described by Burket [1] in 1936. The diagnosis was initially met with scepticism by some authors [2] claiming that it was due to undiagnosed trauma during birth rather than a true congenital condition. However, over the years, the evidence began to trickle into the literature and it is now recognised as a separate condition to the acquired forms of paediatric TMJ ankylosis due to trauma and/or infection [3]. To date, the incidence and aetiology are unknown. In a review of 185 cases of TMJ ankylosis, Topazian [4] in 1964 documented only five cases. Paediatric TMJ ankylosis is known for being a complex and challenging clinical condition and its congenital form adds an additional facet of difficulty.

## 2. Case Presentation

**Figure 1:**
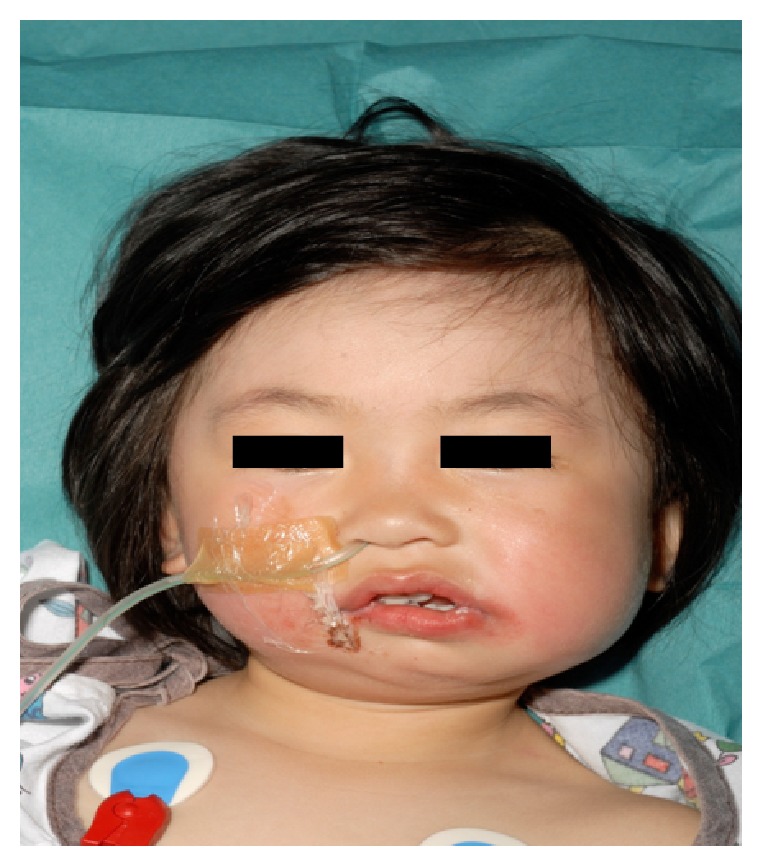
Clinical features at 2 years old.

A 2-year-old female of oriental ethnicity was referred by her paediatrician with the presumed diagnosis of hemifacial microsomia in August 2010. On clinical examination, she demonstrated deviation of the mandible and the chin to the left, lower facial asymmetry, and trismus with a maximal incisal opening (MIO) of 2 mm (Figure 1).

**Figure 2:**
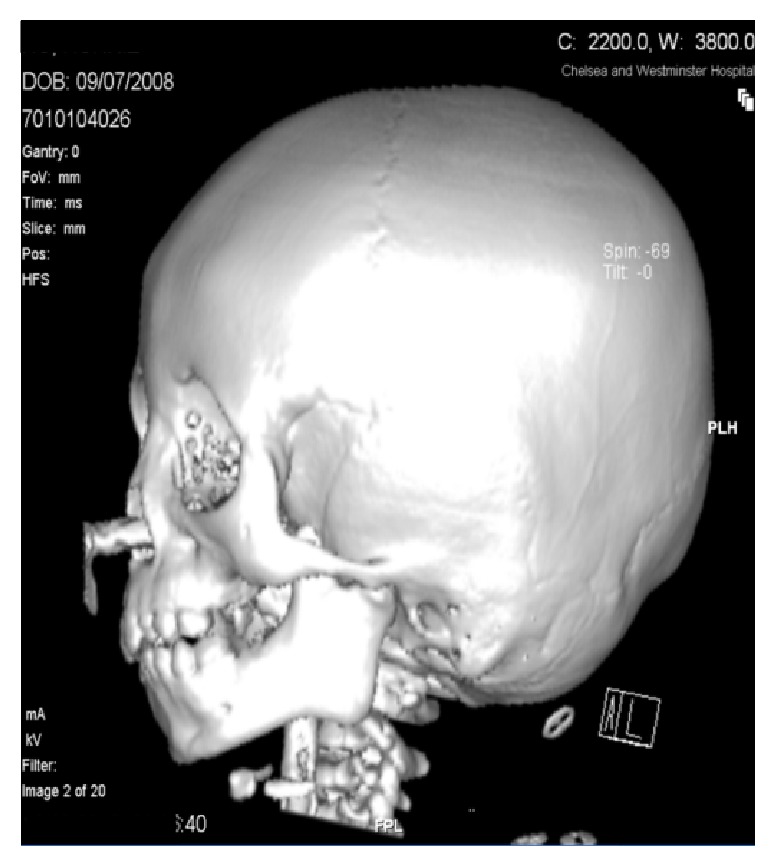
Left view.

**Figure 3:**
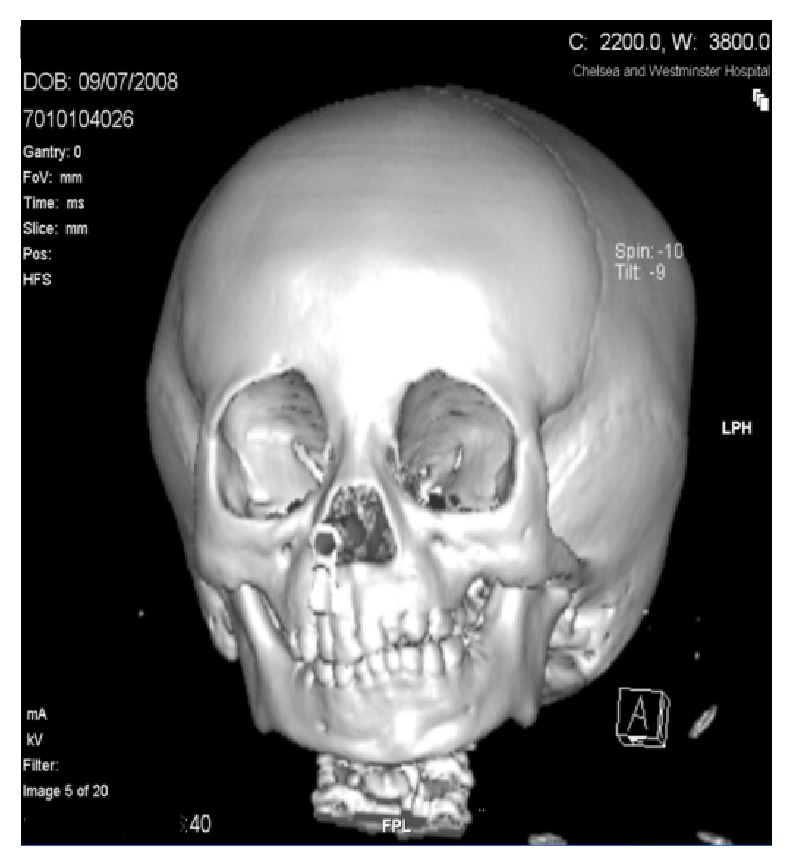
Frontal view.

**Figure 4:**
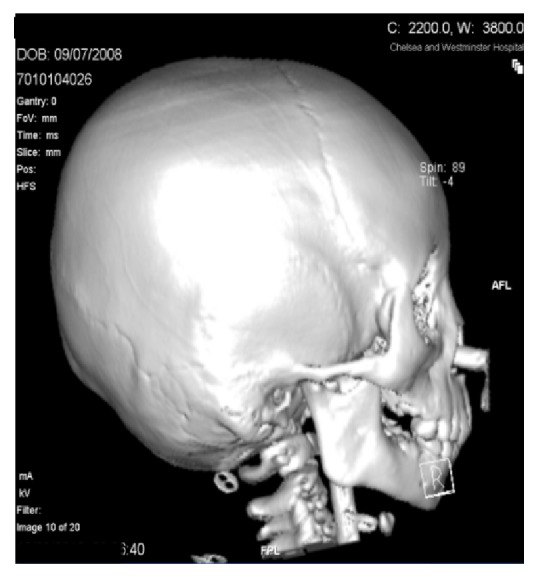
Right view.

**Figure 5:**
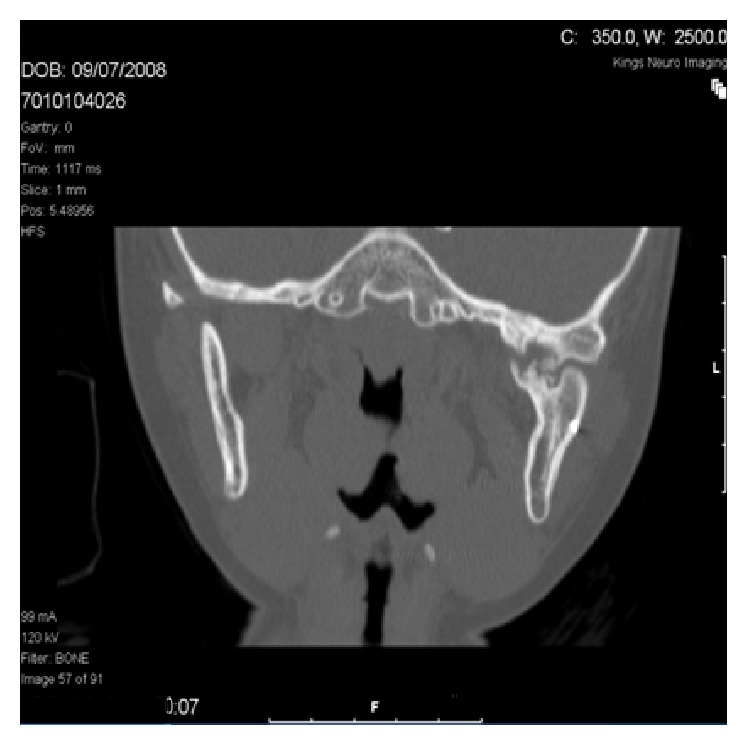
Coronal view of CT scan.

Computed Tomography (CT) scan with 3D reconstruction in 2010 of her jaw under general anaesthesia demonstrated hypoplasia of the left ramus and destruction of the left condyle with ankylosis at the base of the skull (Figures 2 and 4). The left body of the mandible was arch shaped due to restriction of the left condyle with the left coronoid pushing upwards. There was also occlusal cant on frontal CT scan (Figure 3). Coronal view of CT scan demonstrated ankylotic mass (Figure 5).

The patient was born at 39 weeks via spontaneous and natural delivery. Restriction of her jaw movement was confirmed by her dentist in May 2010.

There was no family history of congenital disorders. Her parents are nonconsanguineous.

In May 2011, bilateral intraoral coronoidectomies were performed. Physiotherapy using a wooden spatula was used initially due to lack of access to the TheraBite® Jaw Motion Rehabilitation System*™* (Atos Medical AB).

In June 2011, the patient was seen by a Consultant Geneticist who confirmed that this was a case of isolated developmental abnormality of the TMJ. The patient did not demonstrate any other syndromic features including cleft palate, listening problems, and ear deformities. There was no evidence of trauma or infection during and after birth. Deviation of the jaw to the left and restriction of jaw opening were noticed by her mother soon after birth and during breastfeeding there was significant spillage of milk. She reached all her developmental milestones within the normal time frames.

She was able to feed normally through an open bite deformity on the left.

In February 2012, she underwent excision of the left condylar remnant and costochondral rib graft.

In July 2012, the patient began to utilise the TheraBite® Jaw Motion Rehabilitation System*™* (Atos Medical AB) 3 times a day for 30 minutes per physiotherapy session. The system utilises repetitive passive motion and stretching to restore mobility and flexibility of the jaw musculature, associated joints, and connective tissues. The TheraBite system provides patients with anatomically correct jaw motion. It also helps reduce patients' anxiety by allowing them to control the extent and length of each stretch. The mouth pieces are placed between the teeth. The lever is then squeezed till the point of resistance and held. The mouth is then closed slowly.

In September 2012, she underwent bilateral release of the pterygomasseteric slings and temporalis from the residual coronoid processes. She also had release of scarring from the right TMJ capsule.

In October 2012, she underwent stretching of the jaw under anaesthesia.

Despite increasing physiotherapy on the TheraBite® Jaw Motion Rehabilitation System*™* (Atos Medical AB) to 4 times a day for 1 hour per session, her MIO reduced to 2 mm eventually.

In January 2013, the patient had another CT scan. There was evidence of a bony ankylotic mass obstructing the left TMJ.

In May 2013, excision of the bony ankylotic mass at the left TMJ was performed and an intraoperative MIO of 25 mm was achieved. Physiotherapy commenced 3 days postoperatively on the hospital ward. The teachers at school were requested to help with the physiotherapy sessions.

At follow-up session in July 2013, her MIO was maintained at 21 mm. She was able to engage in daily activities of mastication and speech without any functional difficulties.

## 3. Discussion

A literature review was performed using electronic databases (PubMed, Medline) with the keywords “congenital”, “paediatric”, “temporomandibular”, “joint”, and “ankylosis” and manual cross-referencing between the literatures. This yielded 11 manuscripts published reporting specifically on the management and outcomes of CTMJ ankylosis in the English literature.

The outcomes reported in the literature reflect our own experience of having recurrence of the ankylosis and having to perform a number of operations to improve and maintain the maximal incisal opening of our patient. Shamia et al. [5] have observed noncompliance to jaw opening exercises in the congenital form as a major cause of recurrence.

The earlier in the development stage the TMJ ankylosis occurs, the stronger and more apparent the future maldevelopment of the mandible is [6]. Wittbjer et al. [7] have long term roentgen stereometric data to support this. In that context, the congenital form of TMJ ankylosis is right at the extreme end of the spectrum when it comes to how early the TMJ ankylosis occurs.

Patients with a traumatic cause receive more satisfactory functional result after surgery compared to patients with a congenital cause [8]. This is due to neuromuscular coordination difficulties and muscular disuse atrophy experienced by patients who have TMJ ankylosis from birth.

A case of aplasia of the right internal carotid artery [9] that occurred with an associated finding of right CTMJ ankylosis could suggest vascular disruption during embryological development as a cause. Based upon a single familial case report describing siblings of different sexes with no history of parental consanguinity or description of associated anomalies, a genetic form has been suggested [10].

Gap arthroplasty, interpositional arthroplasty, and osteotomy across and excision of the ankylotic mass within the TMJ have all been described. Kaban et al. recommended the use of transport distraction osteogenesis or costochondral graft and rigid fixation to reconstruct the ramus-condyle unit in TMJ ankylosis patients [11]. The benefits of a costochondral graft include its growth potential, its biologic compatibility, and its capacity to remodel into a neocondyle with time. Its major drawbacks are donor site morbidity and reported unpredictable growth. The greatest advantage of the transport distraction osteogenesis technique is that the patient is able to open and close their mouth and masticate during the process of neogenesis of the condyle, which occurs from the patient's own tissue without any donor site morbidity [12]. A major disadvantage is that a growth center is not transplanted. The variety of techniques described in the published data for the treatment of TMJ ankylosis reflects the complexity of the problem.

Therefore, we have 5 key learning points as follows:

1. Congenital temporomandibular joint ankylosis is an uncommon condition that poses additional challenges to the management of paediatric temporomandibular joint ankylosis.
2. Due to the younger age of these patients, a robust physiotherapy programme will be difficult to put in place initially because of noncompliance.
3. There is an added time pressure when managing a patient with the condition as the insult to the TMJ is earlier in the developmental pathway.
4. There is reduced opportunity for utilising and developing the functional muscles of mastication from birth resulting in neuromuscular coordination difficulties. This is a possible explanation for a post-op MIO of 21 mm in the presented case.
5. Key management principles include adequate removal of the ankylotic mass, costochondral grafting, and robust post-op physiotherapy.
